# Market instability and the size-variance relationship

**DOI:** 10.1038/s41598-021-84680-1

**Published:** 2021-03-11

**Authors:** Sergey V. Buldyrev, Andrea Flori, Fabio Pammolli

**Affiliations:** 1 Department of Physics, Yeshiva University, New York, NY 10033 USA; 2 Department of Management, Economics and Industrial Engineering, Politecnico di Milano, 20156 Milan, Italy; 3 Department of Physics, Boston University, Boston, MA 02215 USA

**Keywords:** Socioeconomic scenarios, Applied mathematics, Physics

## Abstract

We show that some key features of the behavior of mutual funds is accounted for by a stochastic model of proportional growth. We find that the negative dependence of the variance of funds’ growth rates on size is well described by an approximate power law. We discover that during periods of crisis the volatility of the largest funds’ growth rates increases with respect to mid-sized funds. Our result reveals that a lower and flatter slope provides relevant information on the structure of the system. We find that growth rates volatility poorly depends on the size of the funds, thus questioning the benefits of diversification achieved by larger funds. Our findings show that the slope of the size-variance relationship can be used as a synthetic indicator to monitor the intensity of instabilities and systemic risk in financial markets.

## Introduction

The assessment of financial stability has become salient in the literature due to both data availability and the number and magnitude of market crashes over the years. Several indicators of vulnerability have been developed at both micro and macro levels to generate system-wide risk evaluations (see, e.g., Refs.^1–4^ among others). Financial systems and the institutions that compose them can be studied as complex networks of interdependencies and interactions in which, on the one hand, individual behaviors affect the system and its fragility and vulnerability to shocks while, on the other hand, the configuration of the system influences the spread of distress through the multiple relationships connecting its participants. These relationships can be analyzed to measure the transmission mechanisms of shocks and the build-up of unstable conditions. In general, market malfunctions can refer to multiple mechanisms such as macro imbalances, market co-movements, information disruptions, negative externalities, and contagion. Although these phenomena have induced the proliferation of several indicators aiming to capture the nature of systemic instability, policy-makers and regulators have tended to focus on a small number of key indicators, with an ongoing effort to develop a few synthetic measures to assess the degree of financial fragility or stress^1^.

In this paper we study financial instabilities through the lens of the mutual fund sector. Mutual funds are financial entities typically operating across several capital markets as professional investors. These funds invest according to a wide spectrum of allocation strategies and the corresponding industry is characterized by the presence of players of different sizes, whose portfolios may present either very concentrated investment positions or well-diversified allocations^5–9^. Given the large amount of assets under management, mutual funds are investigated to understand both their role in the dynamics of financial markets and their contribution in the emergence of some market frictions impacting financial stability at large, such as the presence of heavy tailed distributions in trading volumes and price returns^10–12^.

In recent years, the size and growth of economic entities have been found to depict some fundamental regularities across several economic domains^13–19^. For instance, the Pareto and lognormal distributions are retained as benchmarks to describe the size distribution, while rare events involving either extremely large positive or negative shocks induce growth distributions to be tent-shaped double exponential with power law tails. In addition, smaller entities are likely to exhibit a lower probability of survival than larger ones, but tend to grow faster, while larger entities experience growth rates unrelated to their past performances or sizes. Finally, the variance of growth rates has been found to be higher for smaller entities and to decay with size as a negative power law with an exponent $\beta$ showing a crossover from zero for small sizes to about 0.20 as the size of the entity becomes larger. The universal character of these regularities suggests that they emerge from a parsimonious set of stochastic processes which describe the growth of economic entities as consisting of a large number of units similarly to how scaling laws emerge in statistical physics^20–23^. The set of these regularities provides also discriminatory criteria between several data generating processes^24,25^.

To which extent the above regularities hold also for financial entities is still debated. For instance, some authors^10,26^ suggest a power law for the upper tail of the size distribution of mutual funds, while others^27^ find evidence for a lognormal. Instead, the assumption that financial returns follow a normal distribution has been questioned by empirical evidence in several markets where marginal distributions have shown to display fatter tails than one would expect from a Gaussian, meaning that financial returns can be modelled by a class of large and flexible stable leptokurtic distributions which accommodate for heavy-tailedness and, possibly, skeweness^28,29^. Finally, the relationships between the size and both performance and risk go back in the literature to the early stages of the Modern Portfolio Theory^30–33^, which predicts that riskier assets tend to require higher premia than safer investments and that the diversification extent is function of the correlations among the assets held in the portfolio.

Nevertheless, how the interplay between investors’ behaviours and market dynamics affects the level of heterogeneity of financial entities is a current field of investigation^34–40^. For instance, retail and professional investors, by relying on competing investment styles and different risk appetite profiles, influence the net flow of financial resources, contributing to portfolios turnover and, possibly, impacting on the dynamics of the underlying markets. These aspects are, therefore, crucial for systemic risk assessment and the evaluation of the stability conditions of financial systems. Hence, there is an ongoing research aimed to detect how the size and style of investment funds, with their diversification levels and portfolio illiquidity conditions, shape the vulnerability of the referring financial markets, with relevant implications for the design of appropriate tools and policies to monitor and scrutinize market dynamics and stability.

Against this background, the relationship between the size of mutual funds and the volatility of their growth rates appears as a relevant instrument to study the emerging interactions in the underlying system and, in particular, to assess the heterogeneity of portfolios held by funds as function of their size. In fact, we show that a parsimonious theoretical framework, as the model proposed by Refs.^19,41–43^, can fit the mutual fund sample. For this reason, we refer to such theoretical framework and we propose to use the corresponding slope of the size-variance relationship as a synthetic indicator for evaluating the aggregate conditions of the system arising from the micro-level behaviors of its participants. Specifically, we find that the emergence of the aforementioned market regularities can be reasonably explained by investigating the change in the number and size of the elementary units (namely, stocks). More importantly, our analysis reveals that during the outbreak of financial markets in 2007–2008, the size-variance relationship became flatter. When approaching a critical transition, stocks tend in fact to co-move (see, e.g., Refs.^4,44–47^), thus reducing the diversification benefits that, for instance, larger funds typically provide in tranquil phases. The slope of the size-variance relationship can thus be interpreted as a synthetic indicator of the underlying market conditions and investors’ attitude to risk and diversification. Hence, a lower and flatter slope is informative of the structure of the system, meaning that the heterogeneity of the growth rates poorly depends on the size of the funds. This, in turn, may pose doubts on the extent of the effective portfolio diversification provided by larger funds during crisis phases. The slope of the size-variance relationship can be therefore exploited as a synthetic tool that combines the features of being theoretically micro-founded at fund level and practically relevant for regulators and market participants.

## Results

We refer to the stochastic model of generalized proportional growth proposed in^19,41–43^ to derive the stylized facts in the mutual fund industry. This model is based on two sets of empirically verifiable assumptions regarding the structure of economic entities. The first set is based on the Gibrat’s law of proportional growth of individual units^13^, postulating that sizes of units change according to a random multiplicative process which can be represented by a geometric Brownian motion. The second set of assumptions is the Simon model of preferential growth^15^, according to which economic entities consist of units that can be created with rate $\lambda$ or destroyed with rate $\mu$ in proportion to the number of existing units in the entity; in addition, new economic entities consisting of small number of units can be created with rate $\nu$. In our study, a fund is thus interpreted as an entity formed by its holding assets, whose sizes are measured by the amount of wealth invested to acquire a certain number of their shares. The corresponding market values of these portfolio holdings reflect, therefore, the combined effect of both the decision about the amount of their quantities to be held in the portfolio and the impact of the respective market prices.

Our analysis relies on the CRSP (the Center for Research in Security Prices) Survivor Bias-Free[..] Survivor Bias-Free data set which collects historical records for portfolio holdings and performances of US open-ended mutual funds. Portfolio holdings allow us to filter those funds more involved in specific asset classes (e.g., bonds or stocks). We restrict our analysis to funds which invest predominantly in stocks since they can be more easily mapped in time and across different funds. Other types of assets reflect, in fact, fund-specific exposures (e.g., derivatives) or are dependent on the expiry time of the financial obligation (e.g., bonds), and for these reasons are excluded from our study. In line with Ref. ^27^, this selection is done by choosing those funds with high equity positions corresponding on average to at least $80\%$ in terms of the total net asset value of the fund, i.e. with a prevalence of equity instruments among their portfolio holdings. We refer in the analysis to the CUSIP identifier.

Specifically, for each quarterly reporting date *t* from March 2003 to June 2013, we compute the size of a fund, i.e. *S*(*t*), as the sum $\sum _i^K\xi _i$ of the market values of the *K* holdings of stocks, i.e. $\xi _i(t)$. The market values of these holdings in our sample range between only a few thousand dollars to some billions, which relate to average funds’ sizes of a few billion dollars. The quarterly growth rate of each fund is then computed as $\ln (\frac{S(t)}{S(t-1)})$ and, similarly, for each stock within a fund it is computed as $\ln (\frac{\xi _i(t)}{\xi _i(t-1)})$. We report in Table 1 some summary statistics. Note how our perimeter of analysis tend to enlarge along the sample period, as indicated by the growing number of both funds and the stocks held in their portfolios as well as by the increasing size of the asset under management. Furthermore, the distribution of the size of the funds appears right-skewed with a prevalence of small-sized funds.

**Table 1:** Summary statistics.

Year	N. funds	N. stocks	Avg. fund size (in million $)	Median fund size (in million $)
2003	1615	7379	966.28	203.84
2004	1553	7338	1492.31	214.46
2005	1638	7350	1467.06	261.95
2006	2205	11122	1644.88	266.11
2007	2606	14697	2357.45	306.97
2008	3469	17435	2216.71	260.02
2009	3313	16970	3519.06	298.37
2010	7518	23256	3115.85	360.11
2011	4904	21928	3200.67	379.23
2012	4908	21004	3452.08	400.69
2013	4656	19206	4076.36	465.60

For each year, the table reports: the number of funds, the number of stocks, the average and the median size of the funds, respectively.

In the Supplementary Information, we investigate extensively the aforementioned stylized facts. In general, these stylized facts are typically summarized as: (1) highly skewed size distribution, (2) tent-shaped growth rate distribution in the proximity of the mean, (3) negative size-growth relationship, and (4) negative size-variance relationship. In particular, we provide supporting evidence that funds’ size distribution is likely to follow a lognormal, that growth rates show a tent-shaped distribution, and that the average growth rate of funds is a monotonically decreasing function of size. Instead, this Section focuses on the remaining stylized fact, namely the size-variance relationship.

### The size-variance relationship

The size-variance relationship describes the behavior of the standard deviation of economic entities’ growth rates versus their sizes, which can be roughly characterized as a power law $\sigma _r=S^{-\beta }$ with an exponent $\beta \approx 0.20$ (see, e.g., refs^15,16,18,19,41–43^). In the model proposed by^19,41–43^, the size-variance relationship theoretically follows from the assumption of uncorrelated fluctuations of units and a sufficiently broad distribution of unit sizes.

**Figure 1:**
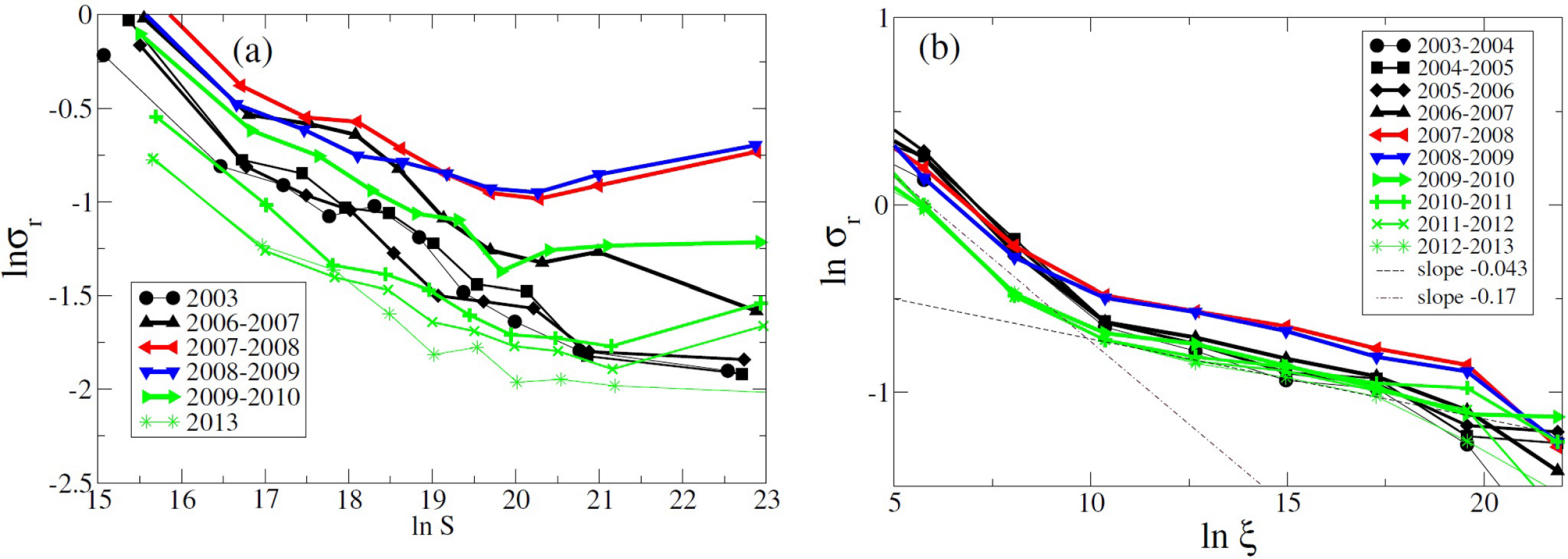
Size-Variance Relationship Log–log plot. (**a**) shows the relationship between the logarithm of the size of the funds and the standard deviation of the growth rates for different time intervals. We divide the entire size distribution in 10 bins each containing the same number of observations. For each bin size, we compute all the growth rates of those funds whose initial market value belongs to that bin and use the standard deviation of the corresponding growth rates as the *y* coordinate on the plot. We also compute the average size of funds in each bin and use it as the *x* coordinate. Log–log plot (**b**) shows the same relationship for the market values of individual stocks.

Figure 1 shows the size-variance relationship for both the market value of funds (see Fig. 1a) and individual stock holdings (see Fig. 1b). One can see that the relationship cannot be very well fitted by a power law, with large funds always fluctuating larger than the power law fitting would predict. In particular, large deviations from the power law behavior are observed for the period of crisis 2007–2008 and the subsequent period of recovery. During these intervals, the volatility of growth rates for large funds becomes significantly higher than that of mid-sized funds. In addition, one can observe the overall increase in volatility during the crisis period for all fund sizes.

Variations in the slope of the size-variance relationship can relate, for instance, to a change in the stock price correlations and/or to a change in the investment behavior of both fund managers and retail investors which modify portfolio exposures. These effects, although ideally distinct, may actually combine with each other and reinforce in periods of market distress, as in the case of fire sales and cascade phenomena. One of the main purposes of our work aims at evaluating the size-variance relationship by proposing a simple distinction between variations occurring due to the impact of market price dynamics and those that refer to changes in portfolios weights. We refer to the latter effect as the investors’ decisions to rebalance portfolios, where variations of portfolio quantities embed the actions of both the retail investors, which may invest in or redeem shares of the funds, and of the fund managers that then actually allocate the available assets under management.

**Figure 2:**
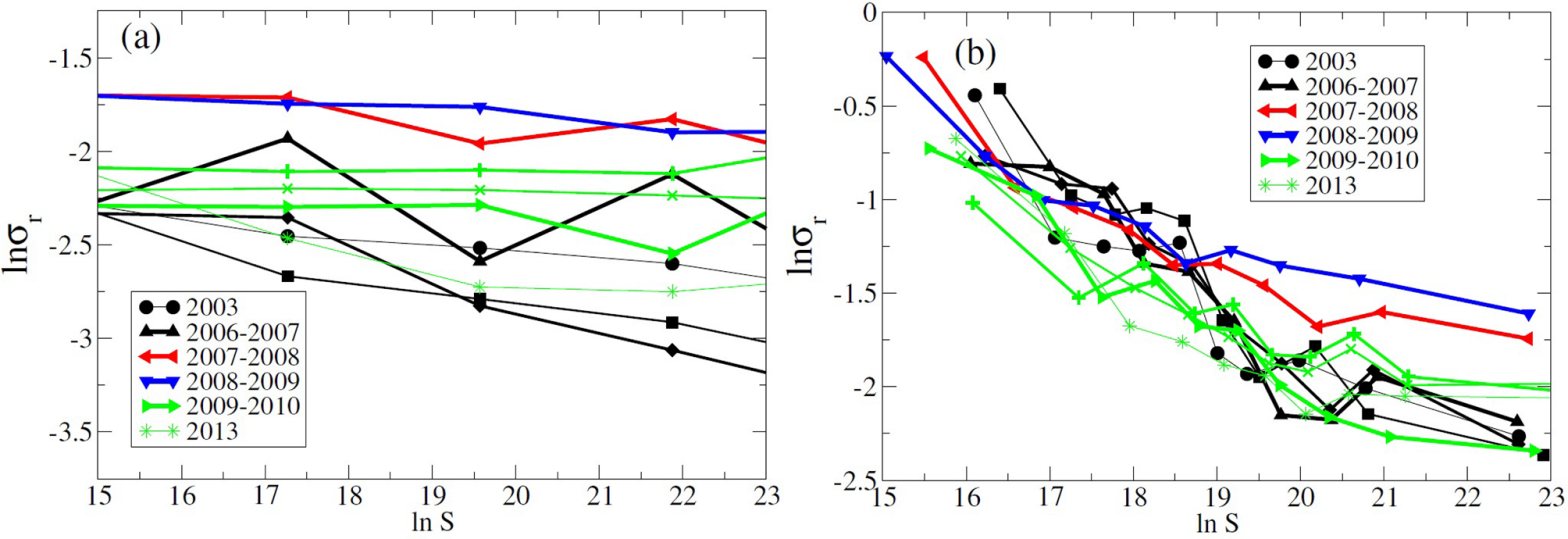
Size-variance relationship for modified data analysis. Log–log plot (**a**) shows the size-variance relationship assuming that market values of stocks vary only due to price changes while the corresponding quantity held in the portfolio is maintained from time $t-1$ to *t*. Log–log plot (**b**) reports the size-variance relationship when all growth events are excluded if at the beginning or at the end of any time quarter, the percentage of stocks in the corresponding fund does not belong to the interval from 98 to 102%.

In order to exemplify the role of investors’ action, we have prepared a synthetic data set in which we leave all the data unchanged, while replacing the market values of the individual stocks at time *t*, namely $\xi _i(t) \equiv n_i(t)p_i(t)$, by $n_i(t-1)p_i(t)$ where $n_i(t)$ is the number of shares of stock *i* and $p_i(t)$ is its price as function of time. We assume, therefore, that market value fluctuations of stocks are caused solely by the dynamics of market prices. The size-variance relationship (see Fig. 2a) computed by this method becomes almost flat, although the overall fluctuations appear again to be much stronger for the crisis years. This example suggests that the investors’ decisions to rebalance portfolio weights contribute to modify the slope of the size-variance relationship, while price movements seem to be responsible for the overall level of the market value fluctuations.

Moreover, a detailed analysis of the growth rates of the largest funds during the crisis years reveals that the increase of variability with size for the very large funds is caused mainly by the actions of a few dozens of funds, which in the apogee of the crisis (in the third and the fourth quarter of 2008) suddenly reduced their holdings in stocks from almost hundred to less than 10%, while in the following quarter they restored the original fraction of the stocks exposure in their portfolios. This action may have the following rationale. Anticipating the continuation of the catastrophic price drop during months subsequent the collapse of the financial markets, these funds may have reduced their market exposure to equity and kept money either in liquidity or in safer assets such as bonds. However, a massive sale of equity assets could have even generated additional detrimental effects on stock prices due to fire sales and cascade effects. It could be the case, therefore, that once the price of stocks dramatically decreased, these funds may have bought them again at a low price in the pursuit of generating high performances when market prices would have eventually rebounded. Obviously, this behavior seems more feasible for larger funds, whose intervention in the allocations might have a relevant impact on stock prices.

**Figure 3:**
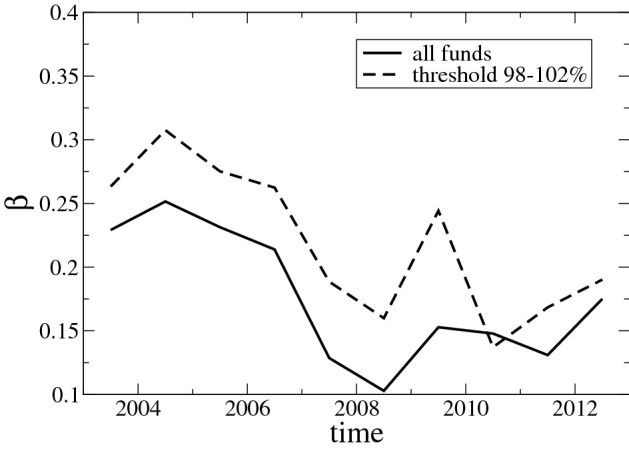
Time dependence of size-variance exponent. ${\beta }$ The values of the slope of the least square fit of the size-variance relationship over the entire range of ln S from 15 to 23 for different years obtained for all observations and only for the observations for which the percentage of stock holdings is between 98 and 102%. The values of the slope fluctuate similarly for both methods reaching the minimum during the crisis and recovery period.

To verify if this behavior can have a real effect, we exclude from the analysis of growth rates those funds whose equity exposure at the beginning or the end of the quarter constitutes less than 98% of the total value of the fund or more than 102% (the percentage of stocks may be greater than 100% due to the presence of liabilities and other asset classes.) Indeed, after removing such observations (see Fig. 2b), the increase of the variance for large funds during the crisis period disappears and the size-variance relationship can be better fitted by the power law $\sigma _r=S^{-\beta }$. The effect of the crisis however is still present in the overall increase of the values of $\sigma$ and the drop in the value of $\beta$ during the crisis period (see also Fig. 3). Since this residual effect can be explained by the increase in the correlation values of stock prices during the crisis, we devote next section to address specifically this point.

### Correlations

**Figure 4:**
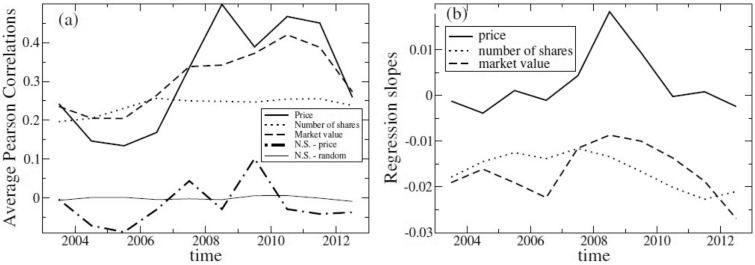
Correlations. Plot (**a**) shows the dependence of average correlations of prices, number of shares and market values of funds’ assets over time and the correlations between the number of shares and the corresponding price changes (N.S.-price) as well as the correlations between the randomized time series (N.S.-random). Plot (**b**) is the dependence of regression slopes of fund sizes for average correlations of the same quantities of Plot (**a**).

One would expect that strong correlations would emerge due to strong co-movements among stock prices. Indeed, the average correlations of price changes among the stocks of the same fund is approximately 0.31, increasing from 0.13 in the pre-crisis year of 2004–2005 to 0.50 during the years of the financial crisis and remaining at large levels even after the crisis (see Fig. 4a). On the other hand, the correlations of the changes of the number of shares have a different behavior, suggesting direct intervention of investors to balance the influence of price correlations on market values. Indeed, the correlations of changes of the number of shares fluctuate around 0.20 and do not change much during the crisis phase. Hence, the correlations of changes of the market values, being a combined result of variations of both prices and number of shares, have the behavior in between their corresponding changes, thus being lower during the crisis of 2007–2008 comparatively to price correlations but increasing with respect to price correlations during the pre-crisis years. To test if the actions of investors relate with price changes, we measure correlations also between price changes and the changes in the number of the corresponding shares for each asset and average them over all funds. We observe that these correlations are negative for the years not affected by the crisis but positive during the crisis. The significance of our findings is confirmed by computing the correlations for the randomized time series and noting that the observed values are several standard deviations far from the randomized ones. This observation suggests that the action of investors may have a stabilizing effect on the market during normal years, meaning that when prices go up they tend to sell and vice-versa, while during the crisis and immediately afterwards the behavior of investors could have reinforced the dynamics of market prices, an aspect which appears coherent with the idea of fire sales and cascade effects during distressed market periods.

With this regard, an important observation is the dependence of price correlations on the size of the fund. During the crisis period, larger funds have higher price correlations between their assets than smaller funds, resulting in a positive regression slope of price correlations and fund size (see Fig. 4b). This finding may suggest the formation during the crisis of strongly correlated groups of stocks, possibly with large autocovariance values (see, e.g., Refs.^44–46^), which are more likely to be present in large funds. Nevertheless, in order to favor diversification, the activity of investors seem to reduce the impact of the correlations of the assets’ price fluctuations by intervening in the proportions of their holdings. This effect appears more pronounced in larger funds and results in reducing and making negative the slopes with respect to the market value of the portfolio exposures.

Therefore, the diversification of portfolio exposures is an important driver for the market dynamics of the corresponding fund. In order to analyze it, we employ the Herfindahl–Hirschman Index (HHI) as a measure of portfolio concentration, computed for each fund using the values of its quarterly holdings. Hence, a value of HHI close to one means that the corresponding fund invests its wealth almost into a single asset, while values pointing to zero stand for more diversified allocation of resources. In particular, the inverse value of the HHI can be interpreted as the number of leading assets in the fund (see, e.g., Ref.^38^). The inverse HHI increases over our sample period, moving from a mean value of about 60 in the years prior to the crisis to almost 75 in year 2009 and above 80 in the post crisis period. Hence, it seems that on average funds opted for less concentrated portfolio allocations in the aftermath of the crisis. Then, we relate the value of the HHI of each fund with its corresponding size by computing the correlation of the logarithmic quantities for each year separately. Our estimates indicate that the correlations move from values around $-0.40$ in the period 2003–2005, decreasing to about $-0.28$ in the biennium before the crisis and reaching the minimum at about $-0.12$ in year 2009, then slightly increasing in absolute value in the subsequent years. Hence, portfolio diversification of mutual funds became less dependent on size during the years of the crisis. This finding is coherent with those reported in Fig. 3, in which the effects of the crisis result in a weakened size-variance relationship signaled by a drop in the value of $\beta$.

## Discussion

The assumption about the absence of correlations between the growth rate of units is clearly violated in the mutual fund industry. In fact, if we identify units as holdings of different stocks, their market values are highly correlated due to correlations among stock prices, especially during crisis years. As a consequence, we would have expected a much weaker size-variance relationship than in other systems in which units are identified as individual products each of which occupies its own independent submarket. Nevertheless, we find roughly the same exponent $\beta \approx 0.16$.

We have shown, in fact, that the size-variance relationship is also affected by the actions of investors. Specifically, we observe that the impact of the changes of the number of shares of stocks held in the portfolios is much greater than the fluctuations in stock prices, dominating the variations of portfolios’ market values. Rather surprisingly, we find that the correlations among stock prices do not play a very significant role in shaping the heterogeneity of the funds’ growth rates. Indeed, we uncover that the abnormal size-variance relationship during the financial crisis is due to the attempts of some large funds to reallocate their equity positions to other asset classes. Interestingly, only a small fraction of funds seems to be engaged in this type of activity in our sample. If we exclude from the analysis all the funds with the fraction of their investment in stocks outside a relatively narrow interval around 100%, then the size-variance relationship during the crisis years restores its approximately power law behavior but with a very low exponent.

From a financial stability perspective, this investigation allows one to reveal important specific features of the system under investigation to be spotted by the variations of the slope coefficient of the size-variance relationship. Hence, we propose to use it as a novel synthetic indicator which exploits the feature of being theoretically micro-founded in order to be practically relevant for regulators and market participants interested in monitoring periods of market instability. In particular, regulators may use the size-variance relationship to provide a system-wide evaluation of stability conditions. A lower slope of the size-variance relationship signals, in fact, a system in which the heterogeneity of the growth rates poorly depends on the size of the funds. This means that in these circumstances the benefits of portfolio diversification, typically provided by larger funds in tranquil periods, appear weakened. A flatter slope is thus informative of the potential sources of vulnerabilities in the underlying system. Instead, fund managers may use this synthetic information to identify their positioning in the size-variance diagram and assess the extent of their effective portfolio diversification, especially in periods of market distress.

There are some extensions that can be considered in future works. Although the use of CRSP is widely diffused in the literature, this sample has some limitations (see, e.g., Ref. ^48^), thus motivating the need to extend the analysis to other data sources, possibly including other capital markets with a globally distributed geographical coverage. Also, a deeper analysis may consider the role of funds with different structural profiles, distinguishing for instance between domestic and international players, or between active versus passive funds. Finally, from a financial stability perspective it would be very interesting to test the predictions of this framework over a longer time horizon and, possibly, with a higher frequency of observation.
